# Supplementary material for: Vertical GeSn nanowire MOSFETs for CMOS beyond silicon
Source: Commun Eng. 2023 Feb 25;2:7. doi: 10.1038/s44172-023-00059-2 (PMC10955907; doi:10.1038/s44172-023-00059-2)
Supplement: Supplementary file 2 — Supplementary Information [file 44172_2023_59_MOESM2_ESM.pdf]

# – *Supplementary Information* –

Vertical GeSn Nanowire MOSFETs for CMOS Beyond Silicon

Mingshan Liu[1], Yannik Junk[1,2], Yi Han[1], Dong Yang[1,2], Jin Hee Bae[1], Marvin Frauenrath[3,4], Jean-Michel Hartmann[3,4], Zoran Ikonic[5], Florian Bärwolf[6], Andreas Mai[6], Detlev Grützmacher[1], Joachim Knoch[2], Dan Buca[1], and Qing-Tai Zhao[1]*

[1]Institute of Semiconductor Nanoelectronics, Peter Grünberg Institute 9 (PGI 9) and JARA-Fundamentals of Future Information Technologies, Forschungszentrum Juelich, 52428, Germany.

[2]Institute of Semiconductor Electronics, RWTH Aachen University, 52056 Aachen, Germany.

[3]CEA, LETI, MINATEC Campus, F-38054 Grenoble, France.

[4]University of Grenoble Alpes, F-38000 Grenoble, France

[5]Pollard Institute, School of Electronic and Electrical Engineering, University of Leeds, Leeds LS2 9JT, United Kingdom.

[6]IHP- Innovations for High Performance Microelectronics, Frankfurt (Oder), 15236, Germany

Corresponding author: q.zhao@fz-juelich.de

1. Material growth and characterization

Ge and GeSn/Ge heterostructures are grown by reduced pressure chemical vapor deposition (RP-CVD) in an industrial reactor. Germane, GeH_4, is used as precursor gas for the Ge-epitaxy and, digermane, Ge_2H_6, and tin-tetrachloride $SnCl_4$ precursors for GeSn epitaxy. The stoichiometry extracted from Rutherford backscattering spectrometry (RBS) is used as an input parameter in the XRD to determine the lattice strain in both Ge and GeSn layers.

The cross-sectional TEM image in Fig. S1(a) shows the 60 nm GeSn layer on a Ge virtual substrate. The high-resolution (HR) TEM image (Fig.S1b) show the high crystalline quality of the $Ge_{0.92}Sn_{0.08}$ layer. The reciprocal space mapping (RSM) (Fig.S1c) show that the peaks of GeSn and Ge lie along the same in-plane lattice vector, confirming the GeSn layer is fully strained. The doping p^+-p^-- p^+ layer stack, needed for p-FET is confirmed by the ECV measurement (Fig.S1d).

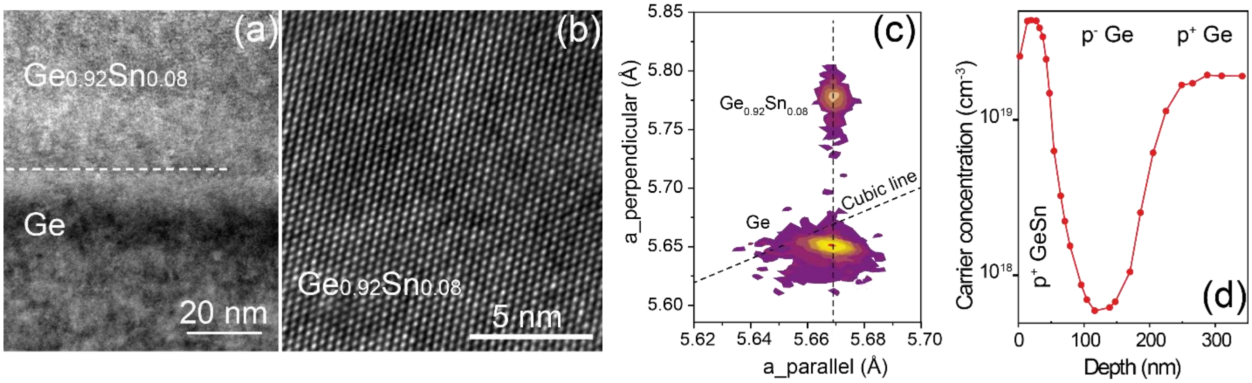

Figure S1*. *GeSn/Ge layer characterization. *(a) Cross-sectional transmission electron microscopy (TEM) image of the $Ge_{0.92}Sn_{0.08}$/Ge interface, showing the absence of defects. (b) A high-resolution-TEM image for the $Ge_{0.92}Sn_{0.08}$ layer confirming the high crystal quality of the grown layer. (c) Reciprocal space mapping (RSM) of an as-grown $Ge_{0.92}Sn_{0.08}$/Ge-stack. The peaks of Ge and $Ge_{0.92}Sn_{0.08}$ lie along the same in-plane lattice vector which shows the pseudomorphic growth. (d) Electrochemical capacitance-voltage (ECV) measurement of the $Ge_{0.92}Sn_{0.08}$/Ge-stack. The top and bottom layers show high in-situ B doping levels.*

The as-grown Ge/$Ge_{0.95}Sn_{0.05}$ /Ge heterostructure for n-FETs is shown by the cross-sectional TEM image (Fig. S2a). Both the top Ge layer and the GeSn layer are ~100 nm thick. The good crystalline quality of the pseudomorphic growth of Ge and GeSn layers without the defect-free interfaces is highlighted by the HR-TEM images and it is supported by RSM data (Fig. S2b)

The activated Phosphorus atoms distribution measured with by Electrochemical-CV (ECV, Fig. S2c), shows doping concentrations of ~7×10^{19} and ~2.5×10^{19} cm^{-3} in the top and bottom Ge layers, respectively. The higher n- doping in the top layer is caused by the different growth parameter considering the thermal budget constraints of GeSn alloy. The device channel region is slightly n-type doped due to the memory effect during the growth. From this doping scheme, the devices indeed work in the accumulation-mode, which is desired for Ge(Sn)-based n-MOSFETs.

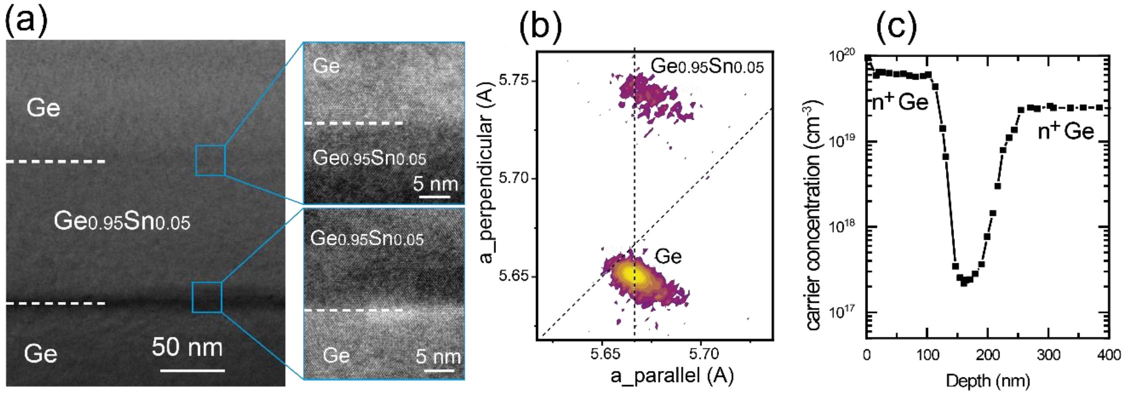

***Figure S2**. **Ge/GeSn/Ge heterostructure for n-VFET characterization**. (a) Transmission electron micrograph of the Ge/$Ge_{0.95}Sn_{0.05}$/Ge epi-stack. The insets show higher resolution images of the Ge/$Ge_{0.95}Sn_{0.05}$-interfaces and demonstrate the absence of dislocations. (b) Reciprocal space mapping (RSM) of as-grown Ge/$Ge_{0.95}Sn_{0.05}$/Ge layers. The GeSn layer has the same in-plane lattice vector as the top and bottom Ge layers, showing the pseudomorphic growth. (c) Electrochemical capacitance-voltage measurement of the Ge/$Ge_{0.95}Sn_{0.05}$/Ge epi-stack. The in-situ P doping is clearly shown in the top and bottom Ge layers.*

2. Band structures

***Table S1**. Calculated band energies for as grown strained GeSn and Ge layers.*

Sn content (at.%)	Ev_{HH} (eV)	Ev_{LH} (eV)	E_{Γ}(eV)	E_L(eV)	Biaxial strain (%)
0	0.000	0.000	0.664	0.804	0
0	-0.017	-0.001	0.779	0.651	0.16
0.080	0.058	0.058	0.609	0.628	0
0.080	0.121	0.029	0.725	0.659	-1.06

The band edges for strained $Ge_{0.92}Sn_{0.08}$ and Ge in “bulk form” (as-grown case) are given in the **Table S1** below. The "effective bandgaps" taken as spacing between the lowest conduction band state and the highest valance band state for as-grown $Ge_{0.92}Sn_{0.08}$ are $E_{gap\text{-}\Gamma}$=0.604 eV, and $E_{gap\text{-}L}$=0.538 eV. The band alignment of the GeSn/Ge heterostructure is given in **Figure S3**. For the relaxed case, cubic GeSn is a direct bandgap alloy with the bandgaps $E_{gap\text{-}\Gamma}$ = 0.551 eV, and $E_{gap\text{-}L}$=0.570 eV. In Figure 1 of the manuscript, the band energies along z-axis for a 20 nm diameter $Ge_{0.95}Sn_{0.05}$/$Ge_{0.92}Sn_{0.08}$ NW heterostructure is calculated after considering 0.46 tensile strain in the top $Ge_{0.95}Sn_{0.05}$ layer, and fully relaxed $Ge_{0.92}Sn_{0.08}$ channel layer. For a 20 nm diameter

$Ge_{0.92}Sn_{0.08}/Ge_{0.90}Sn_{0.10}$ NW heterostructure, 0.3 tensile strain in the top $Ge_{0.92}Sn_{0.08}$ layer, and fully relaxed $Ge_{0.902}Sn_{0.10}$ channel layer are used.

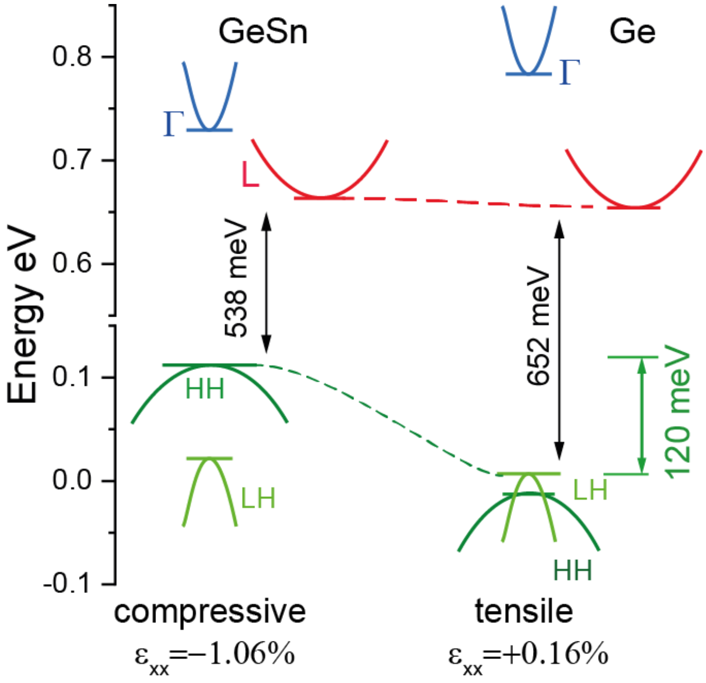

***Figure S3**. Electronic bands alignment for the as-grown GeSn/Ge heterostructure.*

The quantised states of electrons and holes in a quantum wire were calculated within the effective-mass approximation. Mixing of bands near Γ point was neglected. The heavy-hole, light-hole, and the Γ and L conduction bands were described by appropriate effective masses. In the presence of strain these masses are different in different directions. We have used the approximation that the in-plane mass ($m_{||}$) is the same in any direction in the wire cross section, but is different from the mass in the axial, z-direction (m_z). The values of $m_{||}$ and m_z are very different for both heavy and light holes, and also slightly depend on strain, while they are very similar for electrons, as given in **Table S2** for $Ge_{0.92}Sn_{0.08}$, calculated by the *k.p* method. The potential (band edge energy *V(z)*) for the particular carrier type, depends on the material composition and local strain (calculated from the strain value and deformation potentials), as given in **Table S3** for $Ge_{0.92}Sn_{0.08}$. But is here approximately taken to be constant across the wire cross-section, and becomes infinite at the wire surface. It may only depend on *z* in heterostructure wires. Within these approximations the Hamiltonian / Schrödinger equation in cylindrical coordinate system (with *r* the radial coordinate, *z* the axial, and ϕ the azimuthal angle) is

$$-\frac{\hbar^2}{2m_0}\left[\frac{1}{m_{\parallel}}\frac{1}{r}\frac{\partial}{\partial r}\left(r\frac{\partial}{\partial r}\right)+\frac{1}{r^2}\frac{\partial^2}{\partial\phi^2}+\frac{\partial}{\partial z}\frac{1}{m_z}\frac{\partial}{\partial z}\right]\Psi+V(z)\Psi=E\Psi \qquad \text{(S1)}$$

The wave function can then be factorised as

$$\Psi = \mathrm{R(r)Z(z)\Phi(\phi)} \qquad \text{(S2)}$$

with $\Phi = e^{im\phi}$ (m is the azimuthal quantum number) the radial component of energy is given by

$$E_{l,m} = \frac{\hbar^2}{2m_0}\left(\frac{q_{l,m}}{r_0}\right)^2 \qquad \text{(S3)}$$

where $q_{l,m}$ is l-th root of m-th order Bessel function $J_m(q)$=0, r_0 is the wire radius, and l the radial quantum number. The axial component of energy is found by solving the 1D Schrödinger equation by finite-difference method. For single-composition wire of length L the axial component of energy would simply be $E_n = \left(\frac{\hbar^2}{2m_0 m_z}\right)\left(\frac{n\pi}{L}\right)^2$, where n is the axial quantum number.

Table S2. *Calculated effective masses in $Ge_{0.92}Sn_{0.08}$ with biaxial compressive strain.*

ε_{xx}	m_{HHz}	$m_{HH\parallel}$	m_{LHz}	$m_{LH\parallel}$	$m_{\Gamma z}$	$m_{\Gamma\parallel}$	m_{Lz}	$m_{L\parallel}$
0.000	0.222	0.029	0.020	0.050	0.032	0.032	0.117	0.293
-0.002	0.222	0.029	0.022	0.052	0.034	0.032	0.117	0.293
-0.004	0.222	0.028	0.024	0.052	0.036	0.032	0.117	0.293
-0.006	0.222	0.027	0.026	0.050	0.038	0.033	0.117	0.293

Table S3. *Calculated band edges in $Ge_{0.92}Sn_{0.08}$ layer with biaxial compressive strain (reference zero is the valence band. top in unstrained Ge).*

ε_{xx}	E_{HH} (eV)	E_{LH} (eV)	E_{Γ} (eV)	E_L (eV)
0.000	0.058	0.058	0.609	0.628
-0.002	0.071	0.052	0.632	0.635
-0.004	0.085	0.047	0.656	0.642
-0.006	0.098	0.043	0.679	0.650

3. Device fabrication

The fabrication process of vertical Ge/$Ge_{0.95}Sn_{0.05}$/Ge NW n-VFETs is detailed in **Figure S4**.

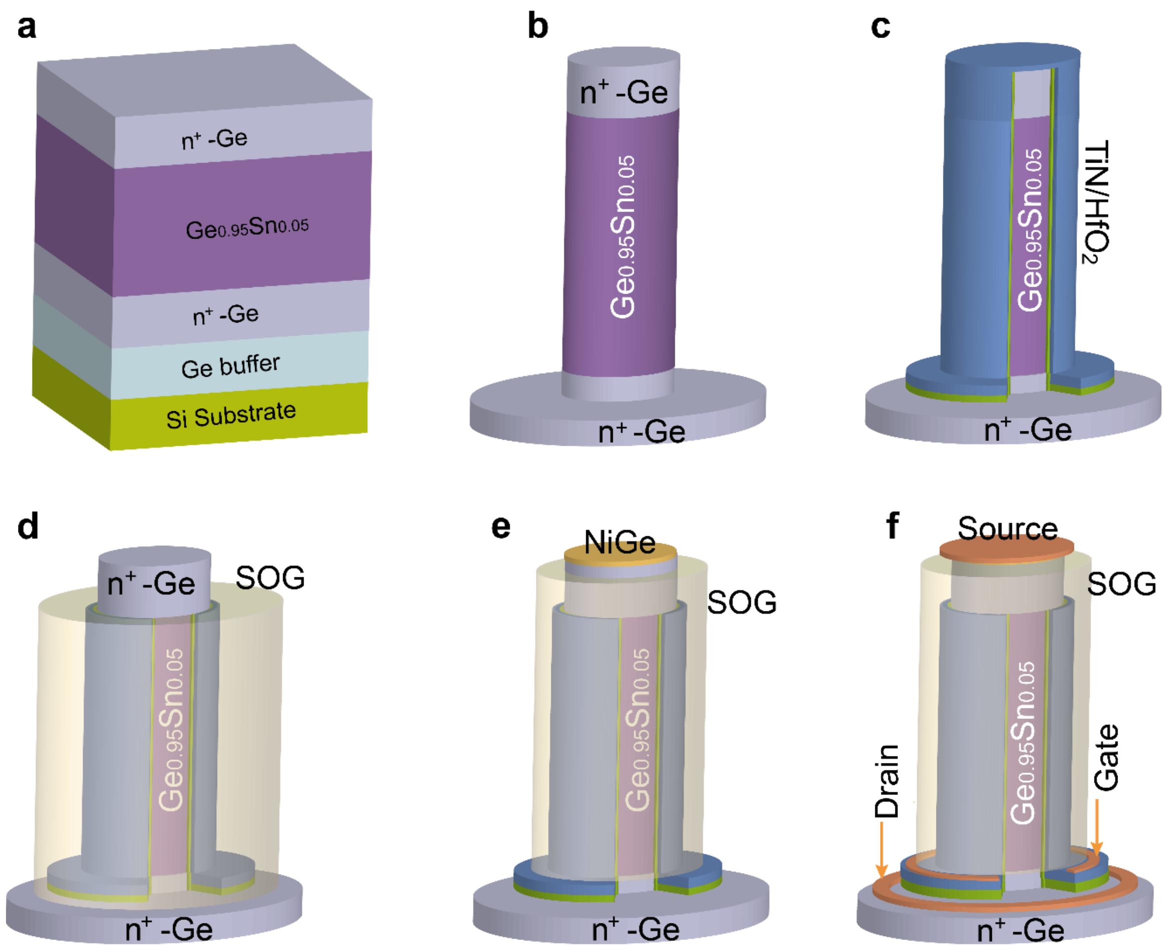

Figure S4**: **Schematic of vertical Ge/GeSn/Ge nanowire (NW) n-MOSFET fabrication process. *(a) layer stack grown by chemical vapor deposition (CVD), (b) Vertical Ge/GeSn/Ge NW fabrication by top-down process; (c) TiN/HfO_2 gate stack deposition and patterning; (d) Spin-on-glass (SOG) planarization and etch back following by gate stack removal from the top of the NW; (e) NiGe formation after SOG planarization to isolate the gate to the top of the NW; (f) metallization of source, drain and gate.*

The vertical NWs are formed using e-beam lithography and RIE etching. Digital etching consisting of multiple cycles of self-limiting O_2 plasma oxidation and diluted HCl stripping is applied to shrink the NW diameters. Subsequently high–κ dielectric deposition starts with an ultra-thin 1 nm ALD Al_2O_3 followed by a post-oxidation process treatment in O_2 plasma. The oxidation passivates the GeSn dangling bonds, forming an ultra-thin $GeSnO_x$ interfacial layer (IL), and saturates oxygen

vacancies in the Al_2O_3 layer. The gate stack is completed by 5 nm ALD HfO_2 and 40 nm TiN deposited conformal around the vertical NWs. Planarization is performed by spin-coated spin-on-glass (SOG) and cured at 350 °C followed by isotropic back-etching with CHF_3. The exposed top gate stack is removed by an optimized Cl_2/SF_6 etching recipe. Subsequently, a second SOG spin-coating and planarization are performed to isolate the gate stack and top contact. NiGeSn is then formed by annealing of a 5 nm thick Ni layer at 300°C. Via openings through the SOG layer are formed and finally Ti/Al contact metallization is conducted to finish the device fabrication. For comparison, Ge control devices with n^+-n^--n^+ doping scheme are also fabricated at the same experimental conditions except the post-oxidation passivation.

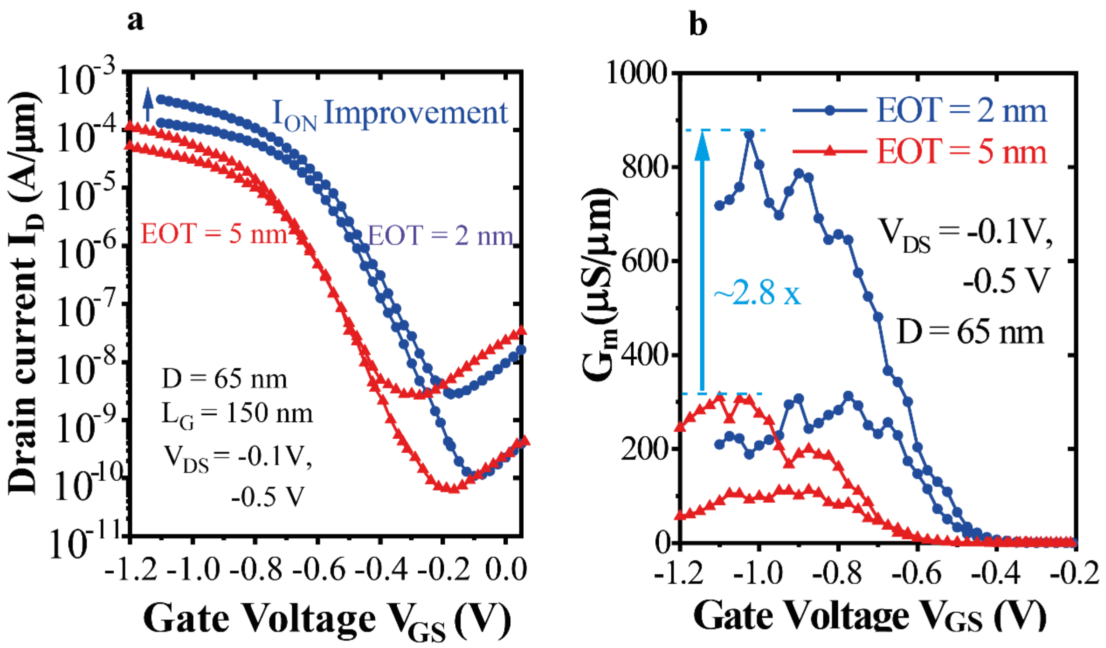

***Figure S5*. Comparison of electrical characteristics between GeSn/Ge and Ge p-VFETs with a nanowire (NW) diameter of D=65 nm.** *(a) Transfer characteristics, showing a subthreshold swing (SS) of 68mV/dec for Ge and 84mV/dec for GeSn/Ge NW devices at* V_{DS}*=-0.1V, (b) Transfer characteristics in logarithmic scale of* I_D *and linear* I_D *(inset) for the 65 nm diameter devices under normalized* V_{GS}*-*V'_{TH}*, showing the on-current increase. (c) Transfer characteristics for p-FETs with a NW diameter of 45 nm further demonstrate the current improvement by using GeSn as source.*

4. *Device performance improvement by EOT scaling*

Few generations of devices were fabricated with two gate oxide thicknesses used 9 nm Al_2O_3 for an equivalent oxide thickness (EOT) of ~5 nm and 5 nm HfO_2 (EOT~2 nm) as the gate oxide for GeSn/Ge p-VFETs, respectively to demonstrate the EOT scaling on the device performance improvement. The transfer characteristics for vertical GAA GeSn/Ge NW p-VFETs with EOT of ~5 nm and 2 nm for 65 nm diameter NWs are compared in **Figure S5 (a)**. The shift of V_{TH} for GeSn/Ge device could be caused by different D_{it} and other process variations. Higher on-currents for EOT=~2 nm are clearly demonstrated in the transfer characteristics. The comparison of the transconductance shows an increasing factor of 2.8 of the peak G_m for the EOT=~2 nm p-VFET (Fig. S5b).

5. *Mobility comparison: The Y-function*

In the linear region with a small drain voltage $V_{DS,}$ the drain current for a MOSFET device is described in the first order as:

$$I_D = \frac{W}{L} \mu_{eff} Q_i V_{DS} \qquad (1)$$

Where *W* and *L* are the gate width (here, the NW perimeter) and channel length, μ_{eff} is the effective mobility, Q_i the charge in the channel. In the first order approximation μ_{eff} can be expressed as [12]:

$$\mu_{eff} \approx \frac{\mu_0}{1+\theta(V_{GS}-V_{TH})} \qquad (2)$$

Where θ is the gate-field mobility reduction factor. The Y- function can be simplified as [1]:

$$Y = \frac{I_D}{\sqrt{g_m}} = \sqrt{\frac{W}{L} C_{ox} \mu_0 V_{DS}} \times (V_{GS} - V_{TH}) \qquad (3)$$

Where $g_m = {\partial I_D}/{\partial V_{GS}}$ is the transconductance of the transistor at a constant V_{DS}. From the Y-V_{GS} linear dependence one can estimate the intrinsic mobility μ_0 of the transistor. For NW transistor we cannot measure the C_{ox} because of the small structure and the unknown dielectric constant of the interfacial layer. We thus estimate the mobility ratio between the GeSn channel and Ge channel n-VFETs from the corresponding Y-V_{GS} line slopes, suggesting that C_{ox} is the same for both devices.

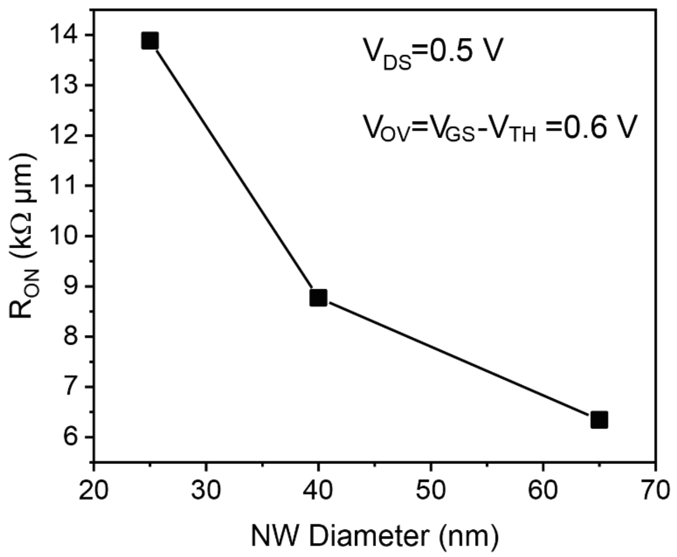

Figure S6. *On-resistance (R_{ON}) as a function of the nanowire (NW) diameter for Ge/GeSn/Ge vertical n-FETs (n-VFETs), showing increasing on-resistance with down scaling of the NW diameter due to higher contact resistance on smaller NW.*

6. All GeSn n-VFET

In order to reduce the off-current induced by GIDL in the low bandgap GeSn drain material as shown in Fig.6b we can use a lower Sn content (<5%) layer at source/drain while use high Sn content layer (>8%) in the channel to improve further the electron mobility (Fig.S6). For lower Sn content source/drain layers it is also much easier to obtain much higher n-type doping concentration to lower further the series resistance.

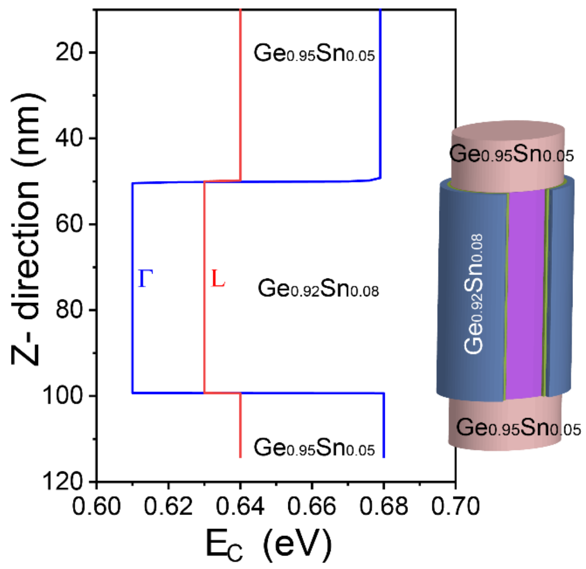

Figure S7*. Conduction band energy alignment for proposed all GeSn vertical n-FET (n-VFET) with direct band channel and highly doped and lower Sn content GeSn alloy as the source/drain.*

7. Cryogenic all GeSn n-VFET

CMOS circuits working at cryogenic temperatures from 100 mK to a few 10 K are required for the control and readout of the qubit information in quantum computing system. In order to reduce the power consumption and the heating dissipation the subthreshold swing SS of a MOSFET should be very small (<5 mV/dec @4 K). However, the interface states and defects cause a band tail in the band, leading to an inflection phenomenon[3] as illustrated in Fig.S7. As a result, SS does not follow the Boltzmann limit and saturates at temperatures T<50 K. The inverse slope S_{inf} above V_{th} becomes much larger, inhibiting the applied voltage V_{DD} scaling.

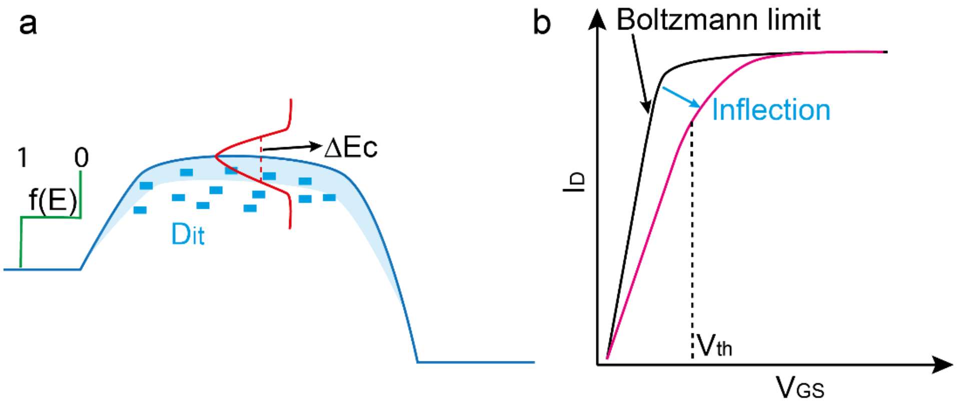

Figure S8. Band tail effects and the inflection phenomenon in the I_D-V_{GS} transfer characteristics. *(a) Schematic illustrating the band tail of the conduction band caused by D_{it} and defects. (b) The band-tail effects result in inflection in the I_D-V_{GS} characteristics, increasing the subthreshold swing (SS) and the inverse slope above V_{th}.*

Figure S8a shows the I_D-V_{GS} transfer characteristics of the full GeSn n-VFET measured at 120 K and 60 K. The decrease of on-currents at lower temperature is caused by high series resistance at source/drain due to relatively low doping and incomplete ionization at low temperature. The SS-I_D characteristics at 12 K show a relative constant SS at I_D <10^{-2} µA/µm and then a sharp increase, following the trend of the ideal curve. We suggest that interface state energies in this low bandgap $Ge_{0.922}Sn_{0.078}$ material are mainly located close to the valence band [4], thus suppressed the inflection phenomenon. Moreover, the electron mobility of the direct bandgap GeSn channel is mcuher higher at low temperatures. All these facts provide a great potential for quantum computing application.

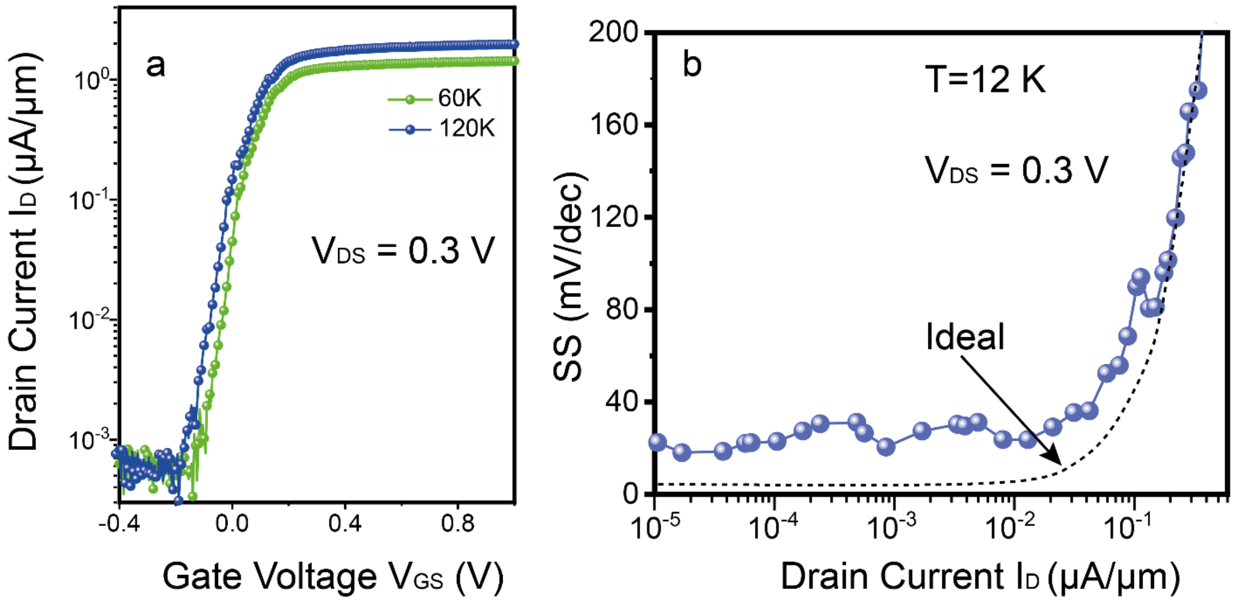

Figure S9. Low temperature characteristics of the full $Ge_{0.922}Sn_{0.078}$ vertical n-FET (n-VFET). *(a) I_D-V_{GS} transfer characteristics the device measured at 120 K and 60 K respectively. (b) SS~I_D characteristics for the GeSn n-VFET measured at 12 K, demonstrating suppressed inflection.*

References

1. Ghibaudo, G. New method for the extraction of MOSFET parameters. *Electron. Lett.* **24**, 543–545 (1988).
2. Henry, J. B., Rafhay, Q., Cros, A. & Ghibaudo, G. New Y-function based MOSFET parameter extraction method from weak to strong inversion range. *Solid. State. Electron.* **123**, 84–88 (2016).
3. Beckers, A., Jazaeri, F. & Enz, C. Inflection Phenomenon in Cryogenic MOSFET Behavior. *IEEE Trans. Electron Devices* **67**, 1357–1360 (2020).
4. Baert, B., Schmeits, M. & Nguyen, N. D. Study of the energy distribution of the interface trap density in a GeSn MOS structure by numerical simulation of the electrical characteristics. *Appl. Surf. Sci.* **291**, 25–30 (2014).
