# Supplementary material for: Vertical GeSn nanowire MOSFETs for CMOS beyond silicon
Source: Commun Eng. 2023 Feb 25;2:7. doi: 10.1038/s44172-023-00059-2 (PMC10955907; doi:10.1038/s44172-023-00059-2)
Supplement: Supplementary file 3 — Description of Additional Supplementary Files [file 44172_2023_59_MOESM3_ESM.pdf]

Description of Additional Supplementary Files

File name: Supplementary Data 1

Description: Source data underlying the graphs and charts presented in the main figures are included in an Excel file.
